# Magnetic resonance imaging-based radiomics analysis of the differential diagnosis of ovarian clear cell carcinoma and endometrioid carcinoma: a retrospective study

**DOI:** 10.1007/s11604-024-01545-z

**Published:** 2024-03-12

**Authors:** Nobuyuki Takeyama, Yasushi Sasaki, Yasuo Ueda, Yuki Tashiro, Eliko Tanaka, Kyoko Nagai, Miki Morioka, Takafumi Ogawa, Genshu Tate, Toshi Hashimoto, Yoshimitsu Ohgiya

**Affiliations:** 1 Department of Radiology, Showa University School of Medicine, 1-5-8 Hatanodai, Shinagawa-Ku, Tokyo, 142-8666 Japan; 2 Department of Obstetrics and Gynecology, Showa University Fujigaoka Hospital, 1-30 Fujigaoka, Aoba-Ku, Yokohama-City, Kanagawa 227-8501 Japan; 3 Department of Pathology and Laboratory Medicine, Showa University Fujigaoka Hospital, 1-30 Fujigaoka, Aoba-Ku, Yokohama-City, 227-8501 Japan; 4 Department of Radiology, Showa University Fujigaoka Hospital, 1-30 Fujigaoka, Aoba-Ku, Yokohama-City, 227-8501 Japan; 5 Department of Radiology, Kawasaki Saiwai Hospital, 31-27 Ohmiya-Tyo, Saiwai-Ku, Kawasaki City, Kanagawa 212-0014 Japan

**Keywords:** Ovarian clear cell carcinoma, Magnetic resonance imaging, Radiomics feature, LASSO algorithm, Texture analysis

## Abstract

**Purpose:**

To retrospectively evaluate the diagnostic potential of magnetic resonance imaging (MRI)-based features and radiomics analysis (RA)-based features for discriminating ovarian clear cell carcinoma (CCC) from endometrioid carcinoma (EC).

**Materials and methods:**

Thirty-five patients with 40 ECs and 42 patients with 43 CCCs who underwent pretherapeutic MRI examinations between 2011 and 2022 were enrolled. MRI-based features of the two groups were compared. RA-based features were extracted from the whole tumor volume on T2-weighted images (T2WI), contrast-enhanced T1-weighted images (cT1WI), and apparent diffusion coefficient (ADC) maps. The least absolute shrinkage and selection operator (LASSO) regression with tenfold cross-validation method was performed to select features. Logistic regression analysis was conducted to construct the discriminating models. Receiver operating characteristic curve (ROC) analyses were performed to predict CCC.

**Results:**

Four features with the highest absolute value of the LASSO algorithm were selected for the MRI-based, RA-based, and combined models: the ADC value, absence of thickening of the uterine endometrium, absence of peritoneal dissemination, and growth pattern of the solid component for the MRI-based model; Gray-Level Run Length Matrix (GLRLM) Long Run Low Gray-Level Emphasis (LRLGLE) on T2WI, spherical disproportion and Gray-Level Size Zone Matrix (GLSZM), Large Zone High Gray-Level Emphasis (LZHGE) on cT1WI, and GLSZM Normalized Gray-Level Nonuniformity (NGLN) on ADC map for the RA-based model; and the ADC value, spherical disproportion and GLSZM_LZHGE on cT1WI, and GLSZM_NGLN on ADC map for the combined model. Area under the ROC curves of those models were 0.895, 0.910, and 0.956. The diagnostic performance of the combined model was significantly superior (*p* = 0.02) to that of the MRI-based model. No significant differences were observed between the combined and RA-based models.

**Conclusion:**

Conventional MRI-based analysis can effectively distinguish CCC from EC. The combination of RA-based features with MRI-based features may assist in differentiating between the two diseases.

**Supplementary Information:**

The online version contains supplementary material available at 10.1007/s11604-024-01545-z.

## Introduction

Epithelial ovarian cancer (OC), which accounts for 90% of all cases of OCs, has been divided into five major histopathology subtypes: low-grade serous carcinoma, high-grade serous carcinoma, endometrioid carcinoma (EC), clear cell carcinoma (CCC), and mucinous carcinoma [1, 2]. CCC and EC are the most common types of epithelial OC that are highly associated with endometriosis [3, 4]; however, compared with EC, advanced-stage CCC is associated with a poorer survival rate owing to the resistance of platinum [5, 6]. The 5-year disease-specific survival of patients with CCC is poorer than that of those with EC even after adjusting for stage: 85.3% vs. 92.7% for stage I, 60.3% vs. 81.9% for stage II, 31.5% vs. 50.6% for stage III, and 17.5% vs. 34.6% for stage IV [7]. Fertility-sparing surgery (FSS) may be considered for patients with stage IA non-CCC of low histological grade (i.e., low-grade EC) who wish to preserve fertility [8]. FSS, which comprises unilateral salpingo-oophorectomy and comprehensive surgical staging, is less invasive than cytoreduction surgery, which comprises hysterectomy/bilateral salpingo-oophorectomy, comprehensive surgical staging, and debulking as needed, in stage IA–IV surgical candidates, regardless of histological subtypes [9]. Therefore, discriminating CCC from EC preoperatively using an imaging modality is a meaningful task.

Magnetic resonance imaging (MRI) has been used to differentiate epithelial OC subtypes based on the morphologic characteristics and signal intensity of tumors [1, 2]. CCC is visualized as a unilocular cystic lesion with polypoid mural nodules more frequently than EC [9]. EC represents as multilocular cystic lesions with large broad-based mural nodules entrapped within the locules [1, 10, 11]. The imaging feature “polypoid growth pattern of the mural nodule” has demonstrated an accuracy of 73.4% for discriminating CCC from EC [10]. Mural nodules have been visualized as hyperintense structures in the T2-weighted image (T2WI) and cystic component on T1-weighted image (T1WI) in CCC and EC [12, 13]. Thus, MRI-based features could be overlapping between the two tumors. The apparent diffusion coefficient (ADC) value (mm^2^/second), a characteristic feature of diffusion-weighted imaging (DWI), is a potential imaging marker related to tumor microstructure [14]. The ADC value of the solid component in CCC is higher than that of the solid component in EC [14, 15]; thus, it could be a useful indicator for predicting CCC.

Radiomics is an emerging field for the post-processing of images and the development of quantification metrics that link qualitative and/or quantitative imaging data for diagnosis, prognosis, and treatment response evaluation [16]. Texture analysis, a form of radiomics, is a quantitative technique that has facilitated the evaluation of the gray-level intensity and the relationship between pixels [17]. The whole OC volume was assessed during texture analysis to differentiate epithelial OCs in previous studies [18–21]. However, no studies have investigated the potential of MRI texture analysis for discriminating CCC from EC [22].

Therefore, this study aimed to evaluate the diagnostic performance of MRI-based features and texture features for distinguishing between the two lesions.

## Materials and methods

### Patients

**Fig. 1:**
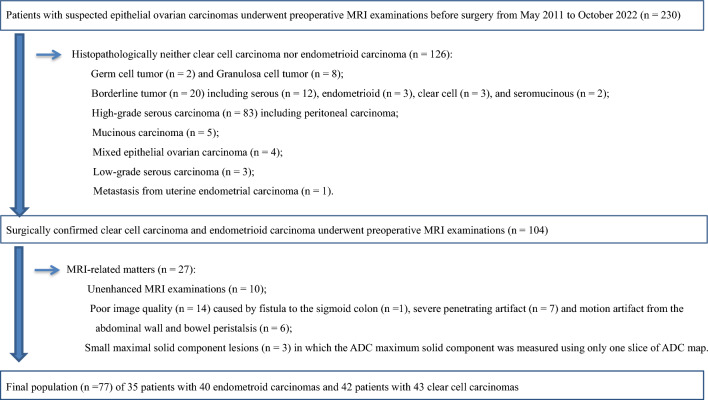
Flowchart of inclusion and exclusion criteria in this study

This retrospective study was approved by the Showa University Ethics Committee. The requirement for obtaining informed consent was waived owing to the retrospective design. A computerized database of radiology reports was searched to retrieve pretherapeutic MRI images of patients suspected of having epithelial OC acquired between May 2011 and October 2022. A total of 230 consecutive patients with pathologically ovarian tumors were retrospectively analyzed. Figure 1 presents the inclusion and exclusion criteria. Seventy-seven patients (mean age 54.2 ± 11.5 years; age range, 30–77 years), including 35 patients with EC and 42 patients with CCC, confirmed via salpingo-oophorectomy, were enrolled in this study after the application of the inclusion and exclusion criteria. Cancer antigen 125 (CA125) value, parity, menopausal status, bilaterality, coexistent endometriosis, surgical stage according to the International Federation of Gynecology and Obstetrics (FIGO) criteria in 2014, and pathological grading were recorded as the clinical characteristics (Table 1).

**Table 1:** Clinical and pathological characteristics of the 77 patients

Characteristics	CCC (*n* = 42)	EC (*n* = 35)	*p* value
Age (y), mean ± SD	53.1 ± 11.6	55.0 ± 11.0	0.443
CA125 level (U/mL), mean ± SD	221.7 ± 503.6	929.1 ± 1317.9	< 0.001
Parity			0.339
Nullipara	12 (28.5%)	14 (40.0%)	
Multipara	30 (72.5%)	21(60.0%)	
Menopausal status			0.488
Premenopause	20 (52.4%)	13 (37.1%)	
Postmenopause	22 (47.6%)	22 (62.9%)	
Bilaterality			0.087
Unilateral	41 (97.6%)	30 (85.7%)	
Bilateral	1 (2.4%)	5 (14.3%)	
Coexistent endometriosis			0.248
Presence	19 (45.2%)	11 (31.4%)	
Absence	23 (54.8%)	24 (68.6%)	
FIGO stage			0.459
I	28 (66.7%)	20 (57.4%)	
II	4 (9.5%)	5 (14.3%)	
III	8 (19.0%)	10 (28.5%)	
IV	2 (4.8%)	0 (0%)	
Pathological grading			NA
1		18 (51.4%)	
2		9 (25.7%)	
3		8 (22.9%)	

*EC* endometrioid carcinomas, *CCC* clear cell carcinoma, *SD* standard deviation, *CA125* cancer antigen 125, *FIGO* International Federation of Gynecology and Obstetrics, *NA* not applicable

### MRI protocol

A 1.5-T scanner (Signa HDxt, GE Healthcare, Milwaukee, WI, USA) with a phased-array 8-channel coil was used to acquire pretherapeutic MRI images within 5 months (mean 5.6 ± 3.3 weeks, range, 1–19) before surgery. The images were acquired using the following MRI sequences: a single-shot fast-spin echo (SSFSE) T2WI in the sagittal plane, fast-spin echo T2WIs in the two planes (axial and coronal or sagittal), single-shot spin-echo planner DWI (b = 0, 1000 s/mm^2^) in the axial plane, and dual-echo chemical shift (in- and out-of-phase) T1WIs in the axial or sagittal planes. In addition, unenhanced and multiphase contrast-enhanced T1WIs (0, 30, 90, and 180 s) were acquired in the axial, coronal, or sagittal plane using a three-dimensional liver acquisition with volume acceleration (LAVA) following the intravenous administration of gadoteric acid (Guerbet Japan, Tokyo, Japan) at a dose of 0.2 mmol/kg of body weight and rate of 2 mL/s followed by 20 mL of saline flush with a power injector. An axial LAVA sequence was added 240 s after the contrast-material injection when multiphasic contrast-enhanced T1WIs were acquired in the coronal or sagittal planes. The equilibrium phase was defined as 180- or 240-s acquisition in this study. Table 2 summarizes the scanning parameters for each image. ADC maps were generated from DWIs with b-values of 1000 and 0 s/mm^2^ using the image analyzing system (Volume Analyzer Synapse VINCENT, version 5.1, Fujifilm Medical Systems).

**Table 2:** Parameters of MRI examinations

Sequences	TE (msec)	TR (msec)	Matrix	NEX	FOV (cm)	Slice thickness (mm)	Interval (mm)	FA
Sagittal SSFSE-T2WI	78–82	507–611	320 × 160	0.6	42 × 36	5–7	0.5–1.5	90°
Axial or Sagittal T2WI	121–127	3260–7340	320 × 224	1	27 × 24	5	0.5	90°
Axial or sagittal dual-echo T1WI	2.2/4.4	140–300	320 × 160	1	45 × 40	4–5	0.5	80°
DWI (b = 0, 1000 s/mm^2^)	75–88	5400–8400	192 × 128	6–8	45 × 40	5–6	0.5	90°
Sagittal or coronal LAVA T1WI	1.9–2.3	4.1–4.6	320 × 192	0.6985–0.7125	40 × 36	4 (SP 2)	0	12°
Axial LAVA T1WI	1.8–2.6	3.8–6.5	320 × 192	0.6985–0.7125	40 × 36	4 (SP 2)	0	12

*SSFSE* single-shot fast-spin echo, *T2WI* T2-weighted image, *T1WI* T1-weighted image, *DWI* diffusion weighted imaging, *LAVA* liver acquisition with volume acceleration, *TE* time of echo, *TR* time of repetition, *NEX* number of excitations, *FOV* field of view, *FA* flip angle, *SP* spacing

### Image analysis

The solid component was defined as contrast-enhanced solid tissue comprising a mural nodule and a larger solid portion in the present study [23]. Subtraction images derived from unenhanced and equilibrium phase contrast-enhanced T1WIs were used to verify the solid component boundaries and areas of hemorrhage, fluid, and necrosis [24]. The maximal solid component was defined as the maximal solid tissue within the lesion. Each lesion was described to assess the MRI data qualitatively and quantitatively if masses were present bilaterally.

### MRI-based features

**Fig. 2:**
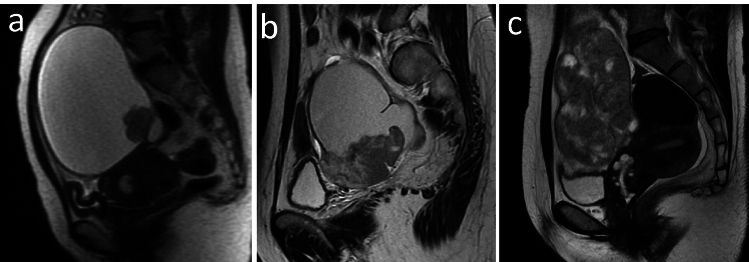
Configuration of the MRI-based features in ovarian carcinomas. **a** Predominantly cystic (≤ one-third solid component) in clear cell carcinoma on single-shot fast-spin echo (SSFSE) T2-weigthed image (T2WI), **b** solid and cystic (one-third to two-thirds solid component) in endometrioid carcinoma on fast-spin echo T2WI, and **c** predominantly solid (≥ two-thirds solid component) in endometrioid carcinoma on fast-spin echo T2WI

**Fig. 3:**
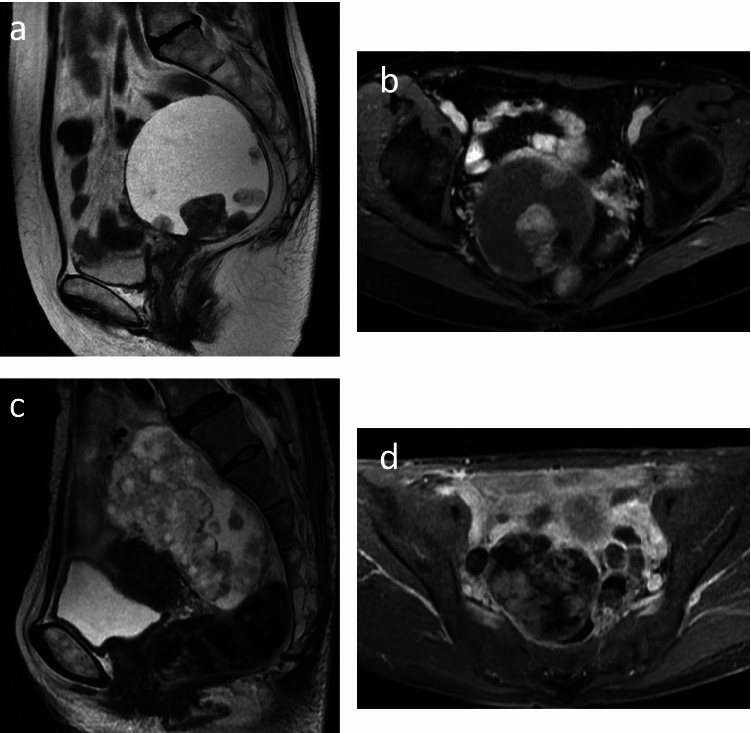
Growth pattern of the MRI-based features in the solid component of ovarian carcinomas. **a**, **b** Polypoid, focal, or eccentric growth pattern of the solid component without continuity in clear cell carcinoma on fast-spin echo (FSE) T2-weighted image (T2WI) and contrast-enhanced T1-weighted image (T1WI). **c**, **d** Broad-based, multifocal, or concentric growth pattern of the solid component with continuity in clear cell carcinoma on FSE T2WI and contrast-enhanced T1WI

Two radiologists (Y.T. and K.N. with 6 and 19 years of experience in abdominal imaging, respectively) who were blinded to the clinical information and pathological results, which were used for inter-reader reproducibility tests, performed qualitative evaluation of MRI-based features independently. Disagreements between the radiologists were resolved by reaching a consensus. The following MRI-based features of OC and the surrounding organs were interpreted initially: (1) morphology (round or oval versus lobulated), (2) configuration (predominantly cystic [≤ one-third solid component], solid and cystic [one-third to two-thirds solid component], or predominantly solid [≥ two-thirds solid component]) [21] (Fig. 2), (3) thickening of the uterine endometrium (defined as an endometrium thickness of ≥ 16 mm and > 5 mm in pre- and post-menopausal women, respectively [25]; categorized as absence or presence), (4) uterine fibroid or adenomyosis (absence or presence), (5) ascites (mild; limited to the Douglas pouch; moderate; limited to the pelvic cavity; and massive, extending beyond the pelvis), (6) peritoneal dissemination (defined as hyperintense nodular, infiltrative or confluent lesions on DWIs with a b-value of 1000 s/mm^2^ and/or contrast enhancement over the peritoneal surfaces, omentum or mesentery; categorized as absence or presence).

The following MRI-based features of the cystic component were interpreted referring to the signal intensity (SI) of the myometrium: (1) SI on T2WIs (hypo-intensity or iso- to hyper-intensity; (2) SI on unenhanced T1WIs (hypo-intensity or iso- to hyper-intensity); and (3) SI on DWI (hypo-intensity or iso- to hyper-intensity).

The following MRI-based features of the solid component were interpreted and evaluated referring to SI of the myometrium: (1) growth pattern including polypoid, focal, or eccentric or large broad-based, multifocal, or concentric [9–11] (Fig. 3); (2) continuity of the mural nodules (defined as a tumor involving more than one-third of the wall; (3) margin of the solid component (smooth or irregular); (4) SI on T2WIs (hypo-intensity or iso- to hyper-intensity; (5) SI on unenhanced T1WIs (hypo-intensity or iso- to hyper-intensity); (6) SI on contrast-enhanced T1WIs during arterial, portal venous, and equilibrium phases (hypo-intensity or iso- to hyper-intensity); and (7) SI on DWI (hypo- to iso-intensity or hyper-intensity).

**Fig. 4:**
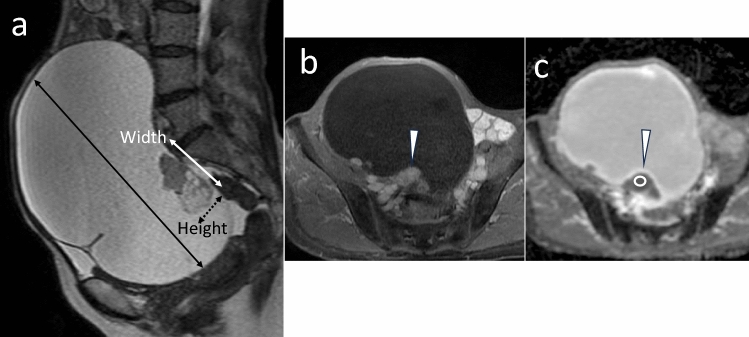
Clear cell carcinoma with FIGOIA in a 66-year-old woman. **a** Sagittal single-shot fast-spin echo T2-weighted image showing the largest dimension with the height and width of the maximal solid component. The size of the maximal solid component was measured: height, 25 mm (**a**, dotted blackline) and width, 49 mm (**a**, white line with bilateral arrowheads). The height–width ratio was 0.510. The maximal lesion size (**a,** black line with arrowheads) was 182 mm. **b** Axial contrast-enhanced T1-weighted image showed solid area (arrowhead) of the maximal solid component. The ADC value (1.54 × 10^–3^ mm^2^/sec) was measured placing a round two-dimensional (50 mm^2^) region of interest (ROI) manually in the solid area of the maximal solid component. Cystic, hemorrhagic and necrotic areas were avoided

The quantitative parameters of the MRI-based features were assessed by two radiologists (N.T. and E.T. with 20 and 22 years of experience in abdominal imaging, respectively). The radiologists were aware of this study’s purpose; however, they were blinded to the clinical information. A third radiologist (Y.T.) selected a single slice in each case that contained the maximal lesion and maximal solid component for each patient. This third radiologist (Y.T.) instructed the two radiologists (N.T. and E.T.) to measure and draw the identified slice. The maximum lesion size (mm) was measured on the sagittal SSFSE- or axial FSE-T2WIs. The height and width of the maximal solid component (mm) at the largest dimension were measured on the sagittal SSFSE-T2WIs with axial or coronal FSE-T2WIs as the reference of subtraction images derived from an unenhanced and equilibrium phase contrast-enhanced T1WIs. The height–width ratio (HWR) (Fig. 4) was calculated using “Height”, representing the maximum vertical length from the bottom of the cyst to the top of the maximal solid component and “Width”, representing the maximum length perpendicular to the “Height” [26].

The ADC value was measured by manually placing a round two-dimensional region of interest (ROI) of 50 mm^2^ on the solid area of the maximal solid component. The cystic, hemorrhagic, and necrotic areas were avoided (Fig. 4). The ADC value was derived by averaging three measurements. Each quantitative variable evaluated by the two radiologists was averaged.

### Texture feature extraction and selection

**Fig. 5:**
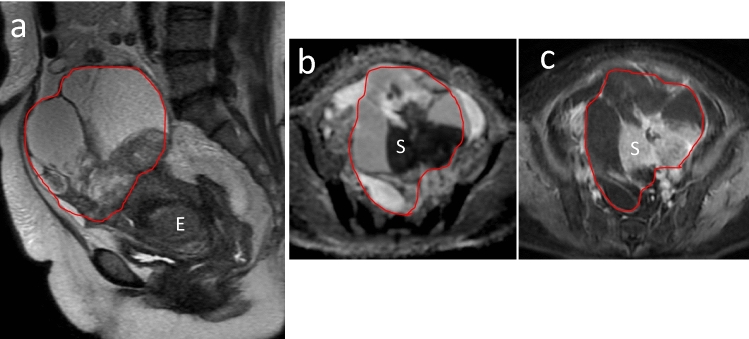
The red volume of interest manually drawn along the margin of the whole tumor in a 56-year-old woman with endometrioid carcinoma. **a** Single-shot fast-spin echo T2-weighted image showing solid and cystic (one-third to two-thirds solid component) tumor. Endometrial carcinoma (E) revealed thickening of the uterine endometrium. **b** Apparent diffusion coefficient map showing hypo-intensity in the solid component (S) within the tumor, indicating restricted diffusion referring to the myometrium. **c** Contrast-enhanced image on equilibrium phase showing hypo-intensity in the solid component (S) referring to the myometrium

Three-dimensional segmentation was performed for a volume of interest (VOI) manually countered along the targeted OC contours on a two-dimensional MRI slice on each sagittal SSFSE-T2WI, axial contrast-enhanced T1WI, and ADC maps (Fig. 5) using LIFEx software (version 7.3.8, French Alternative Energies and Atomic Energy Commission, Paris, France) [27].

Imaging series were imported from a radiomics platform in the Digital Imaging and Communications in Medicine (DICOM) format. The number of gray levels was set at 64 regions of interest (ROI), which were discretized using 64 levels before feature extraction. A random cohort of 30 tumors was segmented by the two radiologists independently (N.T. and E.T.) to assess the reproducibility of the texture features. One radiologist performed the segmentation and the other radiologist confirmed the segmentation for the remaining cases [28, 29]. The interclass correlation coefficients (ICC) were used to determine the inter-reader agreements. Texture features with ICC of < 0.60 and variables with zero variance were excluded from the analysis. The extracted features comprised those that were recommended by the image biomarker standardization initiative [30].

### Statistical analysis

All statistical analyses were performed using SPSS version 26 (IBM SPSS, Chicago, IL, USA) and JMP Pro 17.0.0 (SAS, AS Institute Inc., Cary, North Carolina, USA). A *p* value of < 0.05 was considered statistically significant.

Continuous variables are presented as mean values ± standard deviations, whereas categorical variables are presented as numbers (or percentages). The Chi-square test and Fisher’s exact test were used to evaluate the categorical variables in the univariate analyses to compare between CCC and EC, whereas the Mann–Whitney U test was used to evaluate the continuous variables. All numeric values were standardized using SPSS software before consecutive statistical analysis.

JMP Pro software was used to construct the discriminant models for differentiating CCC from EC. Feature selection was performed using the least absolute shrinkage and selection operator (LASSO) algorithm with a logit link. All variables were included in each feature selection attempt and the LASSO regression coefficients of the variables were obtained via tenfold cross-validation. The maximal number of features was set as four to avoid overfitting. MR-based, radiomics analysis-based, and combined (the best features were selected from MRI features-based and radiomics analysis-based models) models were constructed using this framework. Area under the receiver operating characteristic (AUROC) curves were used to assess the overall discriminant performance of these models. Tenfold cross-validation was repeated 5–15 times during the construction of the final model to decrease optimistic bias in comparing AUROC. The DeLong test was used to compare the differences in AUROC between those models. The accuracy, sensitivity, specificity, precision, and F-measure were calculated based on the classification results.

The consistency of quantitative and qualitative variables between the two radiologists was evaluated using ICC and Cohen’s kappa statistics (k) and categorized as follows: excellent (ICC/k > 0.80), good (ICC/k = 0.61–0.80), moderate (ICC/k = 0.41–0.60), and fair (ICC/k < 0.40).

## Results

### Clinical characteristics of the patients

Table 1 summarizes the clinical characteristics of the 35 patients with EC and 42 patients with CCC. No significant differences were observed between the EC and CCC groups in terms of age, parity, menopausal status, bilaterality, coexistent endometriosis, and FIGO stage. The CA125 value in the EC group was significantly higher (*p* < 0.001) than that in the CCC group. The number of patients with pathological grades 1, 2, and 3 in the EC group was 51.4%, 25.7%, and 22.9% respectively.

### Comparison between the MRI-based features of CCC and EC

No significant differences were observed between the two groups in terms of maximum lesion size, tumor morphology, configuration, uterine fibroid or adenomyosis, ascites, and peritoneal dissemination (Table 3). The absence of thickening of the uterine endometrium was more common (*p* = 0.006) in the CCC group than in the EC group.

**Table 3:** Assessment of MRI-based features in the 83 ovarian cancers

	CCC (*n* = 43)	EC (*n* = 40)	Univariate analysis (*p*)	LASSO estimate	K-value/ICC
Maximum lesion size, mean ± SD	103.9 ± 41.0	98.9 ± 36.2	0.626	0.0000	0.901
(range) (mm)	(38.5–182)	(38–195)			
Morphology			0.231	0.0000	0.820
Round/oval	33 (76.7%)	25 (62.5%)			
Lobulated	10 (23.3%)	15 (37.5%)			
Configuration			0.437	0.0000	0.833
Predominantly cystic	18 (41.9%)	13 (32.5%)			
Solid and cystic	14 (32.5%)	11 (27.5%)			
Predominantly solid	11 (25.6%)	16 (40.0%)			
Thickening of the uterine endometrium			**0.006**	0.2250	0.946
Absence	42 (97.7%)	31 (77.5%)			
Presence	1 (2.3%)	9 (22.5%)			
Uterine fibroid or adenomyosis			0.643	0.0869	0.780
Absence	16 (37.2%)	12 (30.0%)			
Presence	27 (62.8%)	28 (70.0%)			
Ascites			0.092	0.0000	0.922
Mild	35 (81.4%)	24 (60.0%)			
Moderate	7 (16.8%)	13 (32.5%)			
Massive	1 (2.3%)	3 (7.5%)			
Peritoneal dissemination			0.062	0.7003	0.946
Absence	40 (93.0%)	31 (77.5%)			
Presence	3 (7.0%)	9 (22.5%)			

*MRI* magnetic resonance imaging, *EC* endometrioid carcinoma, *CCC* clear cell carcinoma, *SD* standard deviation, *SI* signal intensity, *LASSO* least absolute shrinkage and selection operator, *ICC* intra-class correlation coefficients

No significant differences were observed between the two groups in terms of SI of the cystic component on T2WI and unenhanced T1WI between the two diseases (Table 4). Assessment of the solid component revealed that growth patterns such as polypoid, focal, or eccentric were significantly more common (*p* < 0.001) in patients with CCC than in patients with EC. Broad-based, multifocal, or concentric patterns were more common in patients with EC. The continuity was observed significantly more commonly in EC (*p* = 0.007) than that in CCC. No significant differences were observed between the two groups in terms of margin and SI of the solid component on T2WI, DWI, unenhanced T1WI, and contrast-enhanced T1WI with arterial, portal venous, and equilibrium phases. The mean ADC value of the maximal solid component in CCC (1.40 × 10^–3^ mm^2^/s) was significantly higher than that in EC (1.09 × 10^–3^ mm^2^/s; *p* < 0.001). The height and width of the maximal solid component in the EC group were significantly larger than those in the CCC group; however, HWR did not differ significantly between the EC group (0.67 ± 0.14) and the CCC group (0.69 ± 0.16).

**Table 4:** Assessment of the MRI-based features in the 83 ovarian cancers

	CCC (n = 43)	EC (n = 40)	Univariate analysis (p)	LASSO estimate	k-value or ICC
Cystic component					
T2WI			1.000	0.000	1.000
Hypo- or iso-intensity	2 (4.7%)	2 (2.0%)			
Hyper-intensity	41 (95.3%)	38 (98.0%)			
Unenhanced T1WI			0.122	-0.201	0.927
Hypo-intensity	20 (46.5%)	26 (65.0%)			
Iso- to hyper-intensity	23 (53.5%)	14 (35.0%)			
Maximal solid component					
ADC value, mean ± SD (range) (10^–3^ mm^2^/sec)	1.40 ± 0.18 (1.13–1.87)	1.09 ± 0.19 (0.64–1.67)	<0.001	1.343	0.875
Height, mean ± SD (range) (mm)	29.1 ± 11.7 (12–47)	36.2 ± 15.0 (10–59)	0.027	0.000	0.882
Width, mean ± SD (range) (mm)	43.4 ± 16.9 (16–107)	54.1 ± 19.5 (20–106)	0.017	0.000	0.896
HWR, mean ± SD (range)	0.69 ± 0.16 (0.43–0.97)	0.67 ± 0.14 (0.32–1.05)	0.662	0.000	0.625
Solid component					
Growth pattern			<0.001	0.538	0.953
Polypoid, focal, or eccentric	28 (66.7%)	9 (21.4%)			
Broad-based, multifocal, or concentric	15 (33.3%)	31 (78.6%)			
Continuity			0.007	0.000	0.896
Absence	24 (55.8%)	10 (25.0%)			
Presence	19 (44.2%)	30 (75.0%)			
Margin			0.304	0.000	0.820
Smooth	12 (27.9%)	7 (17.5%)			
Irregular	31 (72.1%)	33 (82.5%)			
T2WI			1.000	0.000	1.000
Hypo- or iso-intensity	2 (4.7%)	1 (2.5%)			
Hyper-intensity	41 (95.3%)	39 (97.5%)			
DWI			0.435	0.000	1.000
Hypo- or iso-intensity	5 (11.6%)	2 (8.0%)			
Hyper-intensity	38 (88.4%)	38 (92.0%)			
Unenhanced T1WI			0.659	0.000	0.975
Hypo-intensity	16 (37.2%)	17 (42.5%)			
Iso-intensity	27 (62.8%)	23 (57.5%)			
Contrast-enhanced T1WI					
Arterial phase			0.340	0.000	1.000
Hypo-intensity	39 (90.7%)	33 (82.5%)			
Iso-intensity	4 (9.3%)	7 (17.5%)			
Portal venous phase			1.000	-0.032	1.000
Hypo-intensity	34 (79.1%)	31 (77.5%)			
Iso-intensity	9 (19.9%)	9 (22.5%)			
Equilibrium phase			0.160	0.000	1.000
Hypo-intensity	27 (62.8%)	31 (77.5%)			
Iso-intensity	16 (37.2%)	9 (22.5%)			

*MRI* magnetic resonance imaging, *CCC* clear cell carcinoma, *EC* endometrioid carcinoma, *LASSO* least absolute shrinkage and selection operator, *ICC* intra-class correlation coefficients, *T2WI* T2-weighted image, *DWI* diffusion weighted imaging, *T1WI* T1-weighted image, *SI* signal intensity, *SD* standard deviation, *ADC* apparent diffusion coefficient

Four features with the highest absolute value of the LASSO regression coefficient were selected to establish a distinguishing model using representative MRI-based features: the ADC value, absence of thickening of the uterine endometrium, absence of peritoneal dissemination, and growth pattern of the solid component (Table 5). The AUC of the prediction model in the MR-based model was 0.895 with accuracy, sensitivity, specificity, and F-measure of 0.831, 0.884, 0.775, and 0.844, respectively. Quantitative and qualitative assessment of the MRI-based features (Tables 3 and 4) revealed that the inter-observer reliability using ICC value was good to excellent ranging from 0.625 to 0.901 and the inter-observer variability using Kappa value was moderate to excellent ranging from 0.780 to 1.000.

**Table 5:** Logistic regression analyses of each individual feature in discriminating clear cell carcinoma and endometrioid carcinoma

	Accuracy	Sensitivity	Specificity	F-measure	AUC
MRI-based features					
The ADC value of the maximal solid component	0.807	0.814	0.800	0.800	0.866
Absence of the thickening of the uterine endometrium	0.614	0.977	0.225	0.724	0.601
Absence of peritoneal dissemination	0.590	0.930	0.225	0.702	0.578
Growth pattern of the solid component	0.711	0.651	0.775	0.700	0.713
Prediction model	0.831	0.884	0.775	0.844	0.895
Radiomics features					
SSFSE-T2WI GLRLM Long Run Low Gray-Level Emphasis	0.590	0.767	0.400	0.660	0.576
cT1WI Spherical disproportion	0.687	0.814	0.550	0.636	0.745
GLSZM Large Zone High Gray-Level Emphasis	0.651	0.884	0.400	0.550	0.640
ADC map GLSZM Normalized Gray-Level Nonuniformity	0.723	0.557	0.900	0.684	0.745
Prediction model	0.843	0.930	0.750	0.818	0.910
Combined model					
ADC value + spherical disproportion and GLSZM Large Zone High Gray-Level Emphasis on cT1WIs + GLSZM Normalized Gray-Level Nonuniformity on ADC map	0.892	0.860	0.925	0.892	0.956

*AUC* area under the curve, *ADC* apparent diffusion coefficient, *SSFSE* single-shot fast-spin echo, *cT1WI* contrast-enhanced T1WI, *T2WI* T2-weighted image, *GLRLM* Gray-Level Run Length Matrix, and *GLSZM* Gray-Level Size Zone Matrix

### Selection of radiomics features for discriminating CCC from EC

Radiomics features with ICC of ≧ 0.6 were included: 98 radiomics features were extracted from each sequence: morphological features (*n* = 12), first-order intensity features (*n* = 19), intensity histogram (*n* = 12), and texture features (*n* = 55) in Supplementary Table 1. The mean ICC value of the 294 radiomics features was 0.961 ± 0.072 (ranging from 0.619 to 1.000) in the inter-observer reproducibility test. All variables were included in the LASSO regression analysis.

Twenty radiomics features with LASSO regression coefficients other than 0 are presented in Supplementary Table 2 and their distribution is presented in Supplementary Fig. 1. In Supplementary Table 2, six variables on the ADC map selected by the LASSO regression algorithm differed significantly between patients with CCC and EC. Table 5 shows that the accuracy (73.5%) of Gray-level Size Zone Matrix (GLSZM) Normalized Gray-Level Nonuniformity (NGLN) on ADC map was superior to those of the other texture features in Gray-Level Run Length Matrix (GLRLM) Long Run Low Gray-Level Emphasis (LRLGLE) (59.0%) on SSFSE-T2WI, spherical disproportion (68.7%), and GLSZM Large Zone High Gray-Level Emphasis (LZHGLE) (65.1%) on contrast-enhanced T1WIs. Four features with the highest absolute values of LASSO regression coefficients were selected for discriminating model using representative radiomics features (Table 5): GLRZM_LRLGLE on SSFSE-T2WI, spherical disproportion and GLSZM_LZHGLE on contrast-enhanced T1WIs, and GLSZM_NGLN on ADC map on ADC maps. The AUC of the radiomics analysis-based model was 0.910 with an accuracy, sensitivity, specificity, and an F-measure of 0.843, 0.930, 0.750, and 0.818, respectively.

### Construction of the combined model

The diagnostic utility of the combined model using the best variables of the MRI-based features and texture features was evaluated. All MRI-based features and radiomics features were included in the LASSO regression analysis. Features with LASSO regression coefficients other than 0 are shown in Supplementary Table 3. The best four features with the highest absolute values of LASSO regression coefficients selected in Table 5 are: the ADC value, spherical disproportion, and GLSZM_LZHGLE on contrast-enhanced T1WIs, and GLSZM_NGLN on the ADC map. The AUC of the combined model was 0.956 with an accuracy, sensitivity, specificity, and an F-measure of 0.892 0.860, 0.892, and 0.925, respectively.

### Comparison of each model

A comparison of the diagnostic performance of the MRI-based, radiomics analysis-based, and combined models revealed that the AUC in the combined model was significantly higher (*p* = 0.02) than that in the MRI-based model. No significant differences were observed between the MRI-based and radiomics analysis-based models (*p* = 0.76), or between the radiomics analysis-based and combined models (*p* = 0.10) in terms of AUCs.

## Discussion

The preliminary study revealed that the diagnostic performance of the combined model, which comprised an MRI-based feature and three radiomics features, was significantly superior to that of the MRI-based model. MRI-based analysis has the potential to be used as a preoperative method to differentiate CCC from EC. The findings of the present study indicate that MRI radiomics analysis imparts an additional value to differentiating between CCC and EC compared with MRI-based visual analysis. To the best of our knowledge, this is the first study to present the results of the MRI-based model and radiomics analysis-based model in this sample.

The growth pattern of the solid component was divided into “polypoid, focal, or eccentric” and “broad-based, multifocal, or concentric” as described in the study by Morioka [9]. Among the pathological findings of CCCs, the growth pattern of “polypoid, focal, or eccentric” in the solid component was associated with the growth pattern of cancer cells such as nests, tubes, and papillae. In addition, CCCs arising from endometriosis forming cysts tend to exhibit a papillary pattern, whereas CCCs arising from adenofibromas tend to demonstrate a tubulocystic pattern; however, the tubulocystic, papillary, and solid architecture often occur concurrently [4, 31]. Consequently, the MRI features of the solid component were diverse, reflecting the diverse tissue structures [10]. In the present study, 66.7% (28/43) of the CCC groups revealed this growth pattern of solid component, which corresponded with the findings of previous studies, which reported this pattern in approximately 60–70% of patients with CCC. A “broad-based, multifocal, or concentric” was observed in the remaining 30–40% of cases [9, 31]. EC can be divided into three grades: high, moderate, and low differentiation according to the configuration of the glands and the degree of differentiation of the tumor cells [32]. Tumor cells with different degrees of differentiation exhibit different growth modes: highly differentiated EC tumor cells exhibit papillary growth, and EC tumor cells with medium and low differentiation proliferate into multiple layers and grow diffusely and continuously [12].

The ADC value of the solid component in CCC was higher than that in EC, which may reflect low cellularity and high extracellular space volume [12, 14, 15, 33]. In this study, mean ADC value (range) was 1.40 (1.13–1.87) × 10^–3^ mm^2^/s in the CCC group and 1.09 (0.64–1.67) with × 10^–3^ mm^2^/s in the EC group, which correspond with previous reports on a 1.5 T MRI scanner with ADC values ranging from 0.98 × 10^–3^ mm^2^/s to 1.51 × 10^–3^ mm^2^/s in patients with CCC and 0.67 × 10^–3^ mm^2^/s to 1.38 × 10^–3^ mm^2^/s in patients with EC [15]. The ADC value of the maximal solid component was the most significant predictor for CCC in the MRI-based model and the combined model (Table 5); however, the mean on the ADC map, as the first-order statistics, was not selected. We believed that variables of radiomics features on the ADC map extracted from the whole tumor volume using VOI were numerically different from those of the ADC value of the maximal solid component using ROI.

Regarding radiomics features, one morphological feature and three radiomics features were selected (four in total). Morphological features describe geometric aspects of an ROI as shape features and the distribution of voxel intensities within the ROI was described as a first-order feature [34]. Spherical disproportion is the ratio of the surface area of the tumor region to the surface area of a sphere with the same volume as the tumor region, and by definition, the inverse of sphericity [35]. In an image, GLRLM quantifies gray-level runs, which are defined as the length of consecutive pixels (reported as the number of pixels) that have the same gray-level value [34]. LRLGLE represents a measure of the joint distribution of long run lengths with lower gray-level values [35]. GLSZM quantifies gray-level zones which are defined as the number of connected voxels that share the same gray-level intensity [36]. LZHGLE is a measure of the proportion in the image of the joint distribution of larger size zones with higher gray-level values [35]. NGLN measures the variability of gray-level intensity values in the image, with a lower value indicating a greater similarity in intensity values. We considered that the pathological difference and architectural heterogeneity might affect the homogeneity of gray-level in three dimensions because CCC had previously shown glycogen-containing clear cells and hobnail cells and EC had demonstrated cylindrical cytoplasm-containing endometrial epithelial cells [4, 32].

It is important to discriminate CCC from EC preoperatively as the survival rate of patients with advanced-stage CCC is poorer than that of patients with EC. FSS may be considered in stage IA low-grade EC; however, the additional value of radiomics analysis in distinguishing them was small because radiomics analysis is a time-consuming process and the MRI-based analysis had sufficient diagnostic potential. A small benefit of MRI texture analysis has been reported in comparing subtypes of ovarian tumors, i.e., epithelial OCs between borderline and malignant tumors, and sex-cord stromal tumors [20, 37–39]. However, further studies must be conducted to identify the differential diagnosis.

HWR did not differ significantly between the two groups during the assessment of MRI-based features; this was inconsistent with the findings of the studies of Morioka [9] and Li [31]. The size limitation for the maximal solid component may have been contributed as 14 lesions with the poor image quality caused by artifacts were excluded despite the presence of large maximal solid components and three lesions that were not sufficiently large to average the ADC value of maximal solid component using two slices of the ADC maps. The frequency of iso- or hyper-intensity of the cystic component, which is attributed to the presence of degenerated blood products owing to co-existing endometriosis, was 53.5% (23/43) in the CCC group. This finding may also correspond with those of the study by Kato (53.7%) [13]. Hypo- or iso-intensity in the solid component on T2WI was only observed in two cases (4.7%) with abundant fibrous tissue in clear cell adenofibroma-associated CCCs (Table 4). This is consistent with the findings of previous studies, which reported that benign or borderline clear cell adenofibroma as a precursor of CCC accounts for < 5% of all clear cell OCs [1, 4, 5].

This study has certain limitations. First, this was a retrospective study with a small sample size conducted at a single center using a single machine vendor; therefore, external validation must be performed using data from other facilities. Second, MR image intensities vary depending on the voxel size, magnetic field strength, pulse sequence, and reconstruction algorithm [40]. Roy et al. reported that features of GLRLM and GLSZM were sensitive to noise, whereas radiomics features, such as kurtosis, were sensitive to changes in resolution on T1WI and T2WIs [41]. Motion and penetrating artifacts within the OCs on each image and the different voxel sizes may influence the radiomics features [40]. Image noise and motion artifacts from the abdominal wall and bowel peristalsis can be decreased by acquiring thin-slice three-dimensional images following the administration of antiperistaltic agents in the prone position. Third, the period between MRI examination and surgery was on average 5.5 ± 3.4 weeks (range, 1–19 weeks). Lastly, three patients with EC who underwent pretherapeutic MRI examinations received neoadjuvant chemotherapy as admission was not permitted in our institution during certain periods of the Coronavirus Disease 2019 pandemic.

## Conclusion

Our preliminary study revealed that the conventional MRI-based analysis is an effective method to distinguish CCC from EC. Radiomics-based features combined with MRI-based features may assist in differentiating between the two diseases. An external validation using data from other facilities with a large patient cohort must be conducted in the future to verify this model.

### Supplementary Information

Below is the link to the electronic supplementary material.

Supplementary file1 (PPTX 2600 KB)

Supplementary file2 (DOCX 23 KB)

Supplementary file3 (DOCX 21 KB)

Supplementary file4 (DOCX 23 KB)
