# Supplementary material for: Magnetic resonance imaging-based radiomics analysis of the differential diagnosis of ovarian clear cell carcinoma and endometrioid carcinoma: a retrospective study
Source: Jpn J Radiol. 2024 Mar 12;42(7):731–43. doi: 10.1007/s11604-024-01545-z (PMC11217043; doi:10.1007/s11604-024-01545-z)
Supplement: Supplementary file 1 — Supplementary file1 (PPTX 2600 KB) [file 11604_2024_1545_MOESM1_ESM.pptx]

## Slide 1
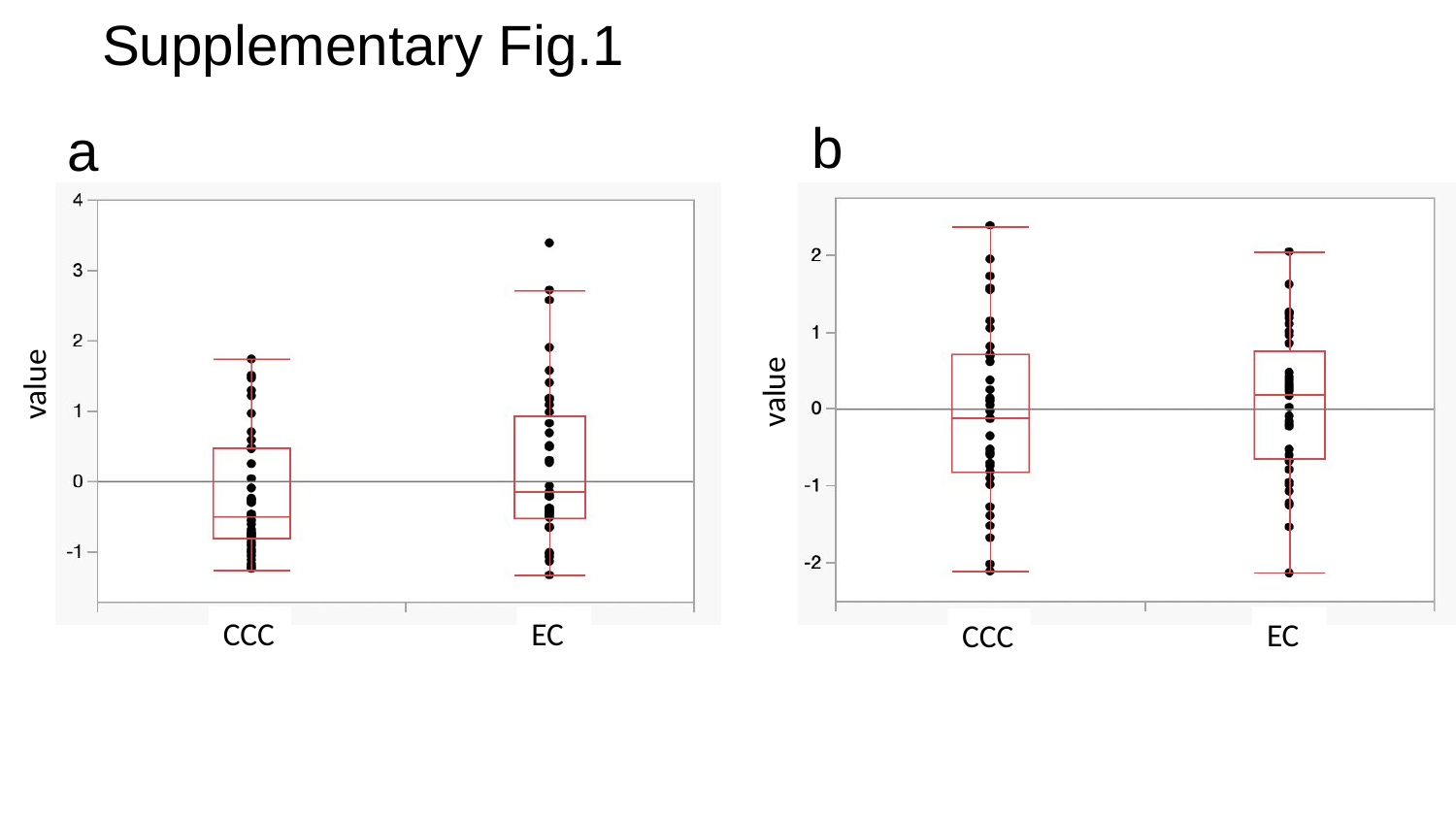

Supplementary Fig.1
b
a
# p@
value
value
CCC
EC
EC
CCC

## Slide 2
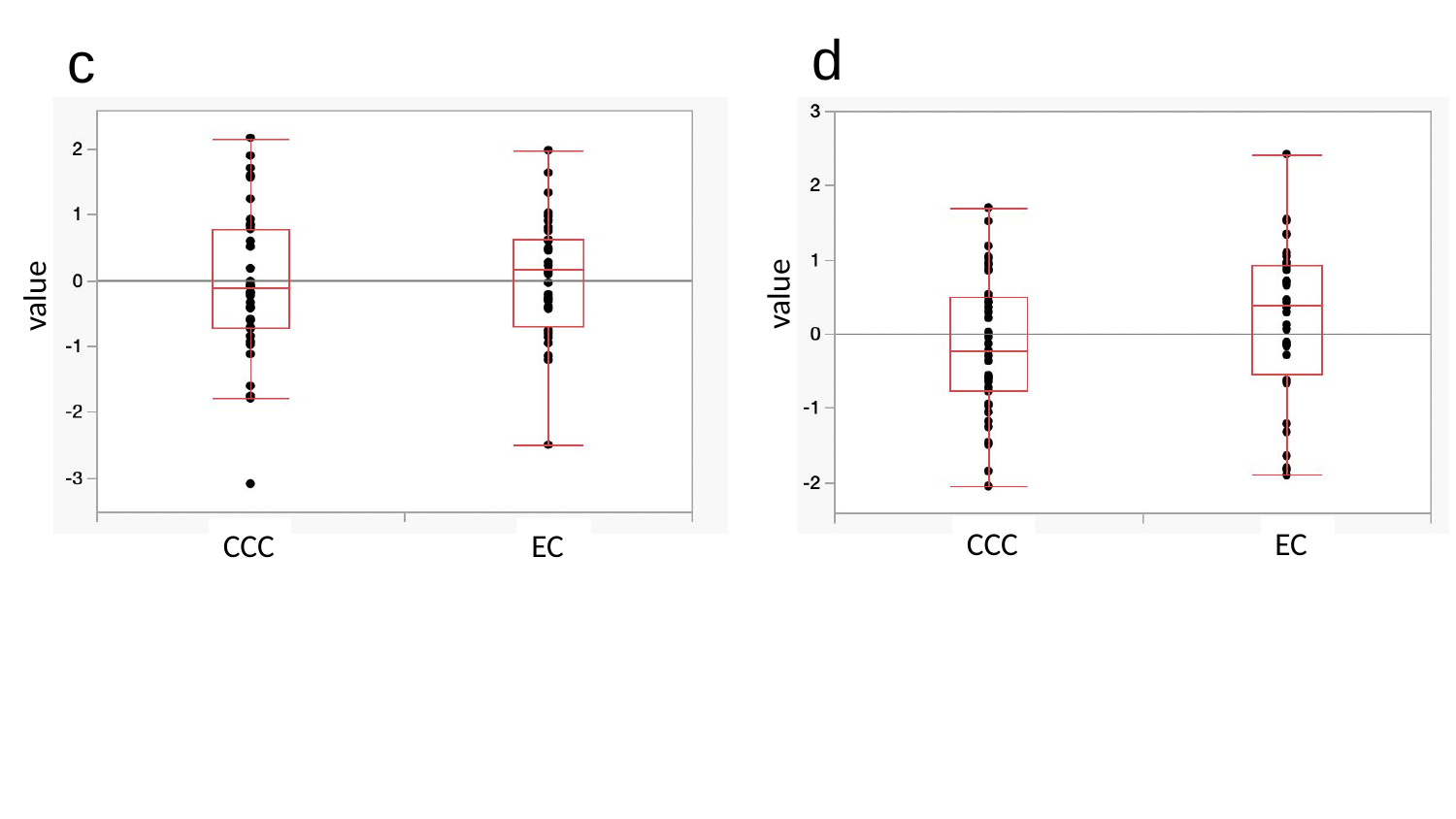

d
c
value
value
CCC
EC
CCC
EC

## Slide 3
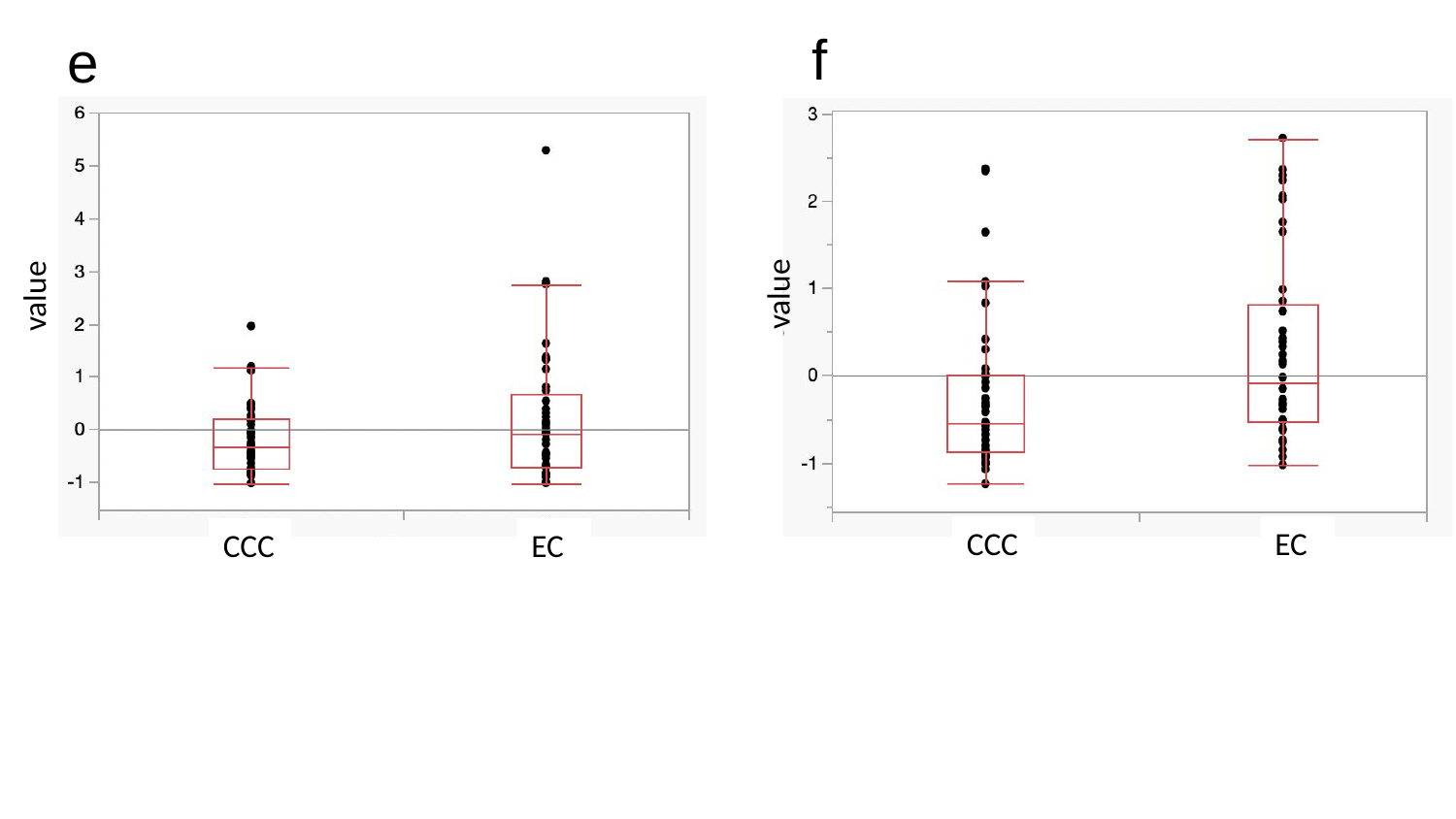

f
e
value
value
CCC
EC
CCC
EC

## Slide 4
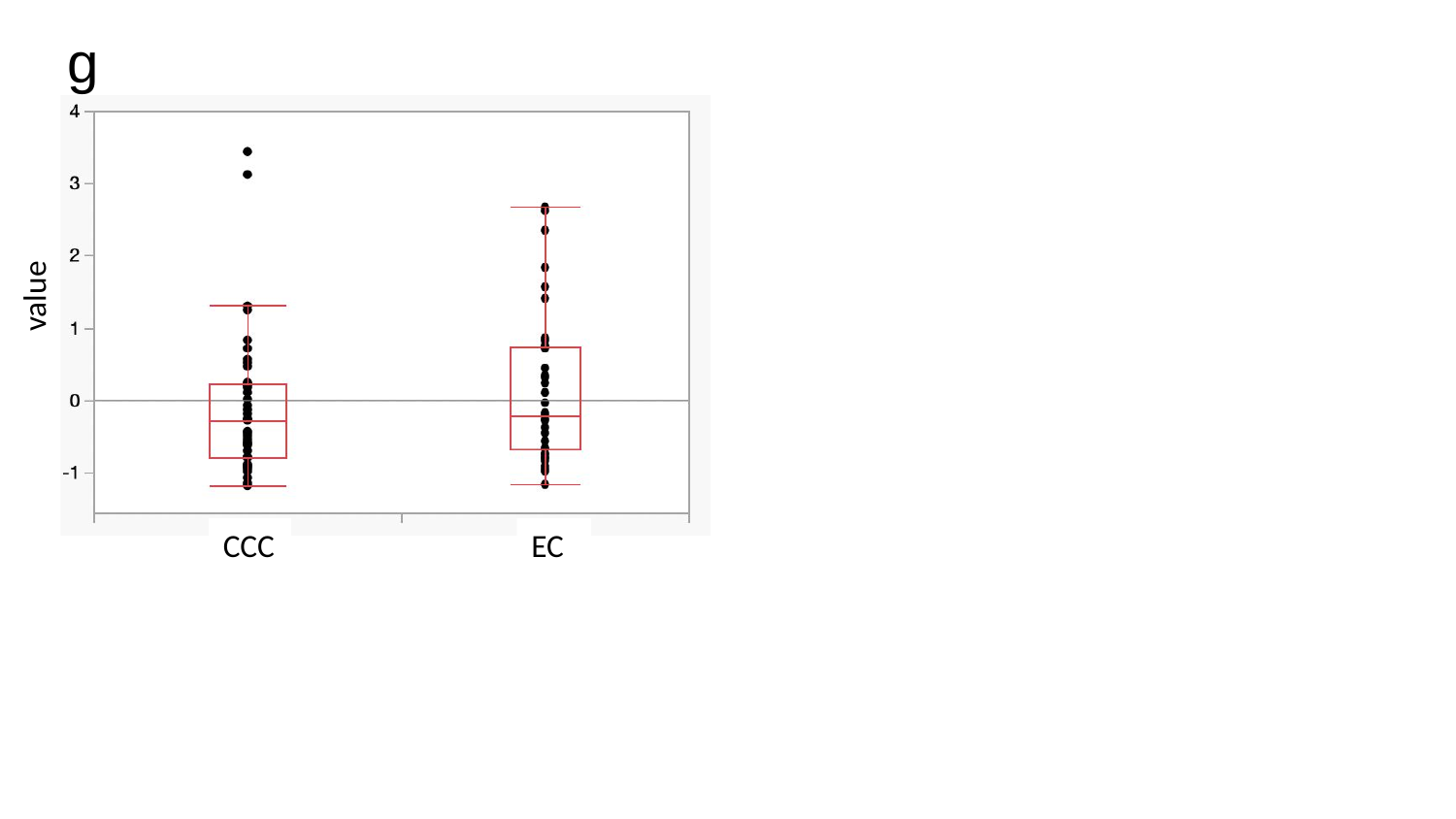

g
value
CCC
EC

## Slide 5
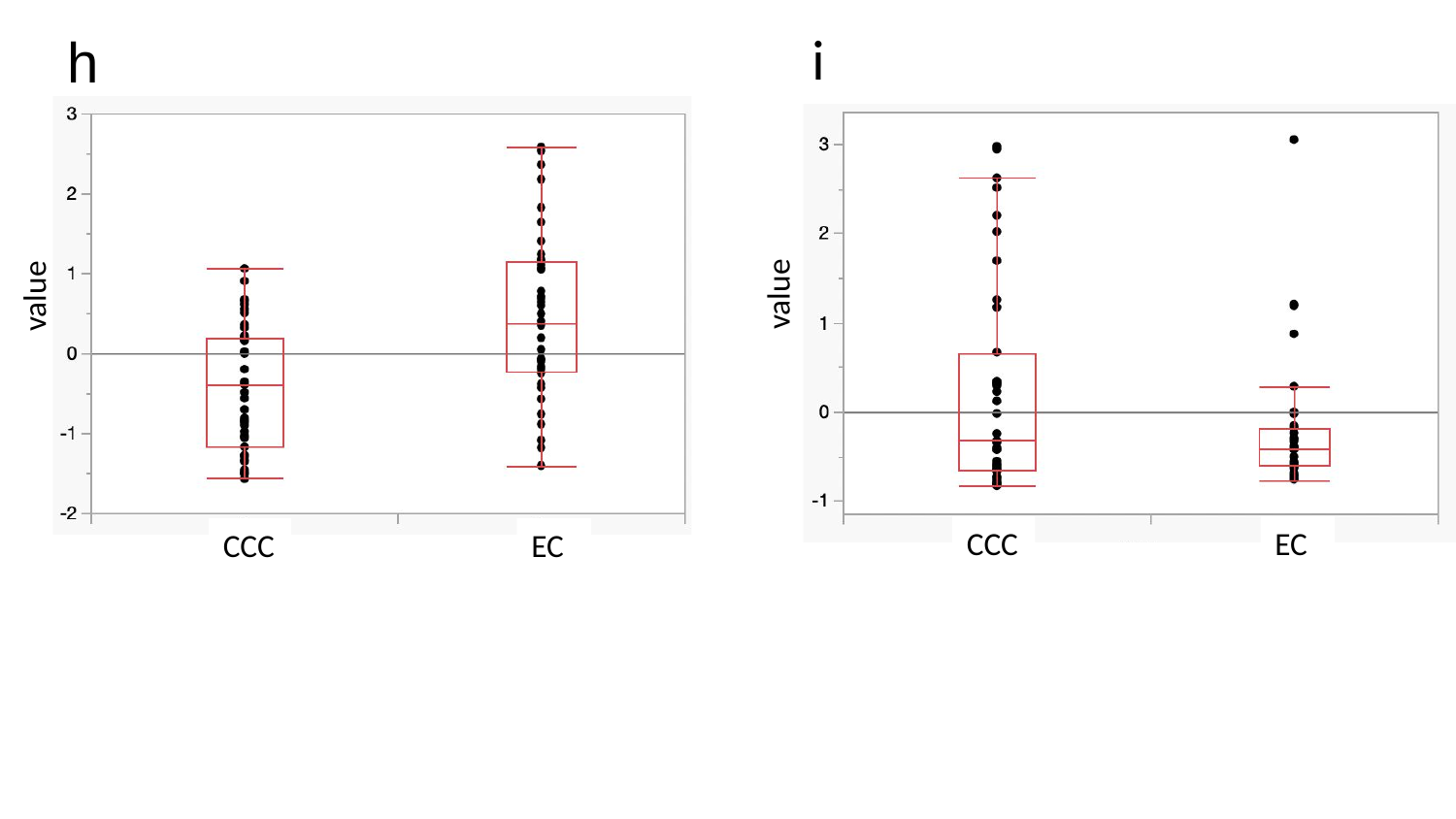

i
h
value
value
CCC
EC
CCC
EC

## Slide 6
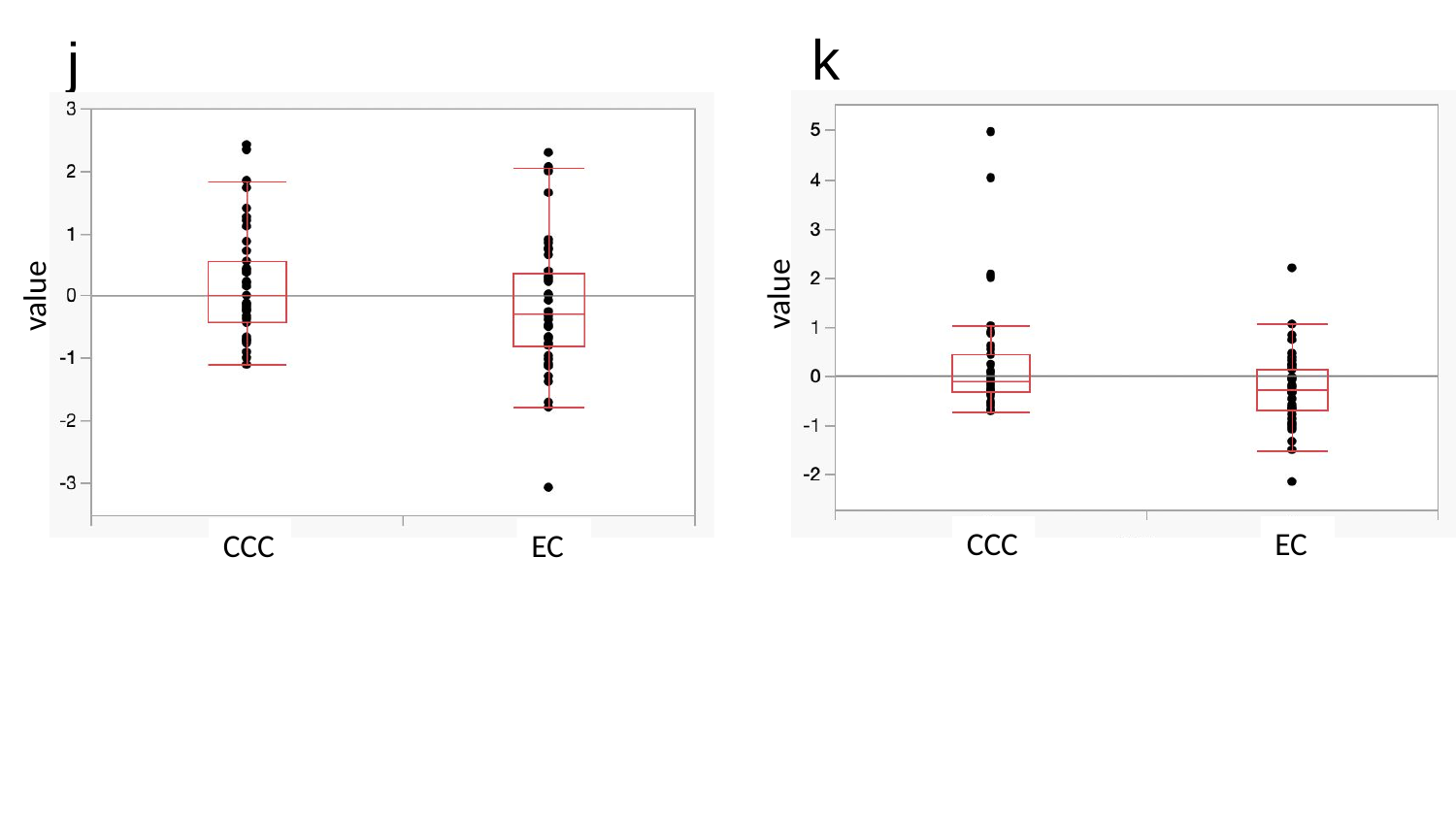

k
j
value
value
CCC
EC
CCC
EC

## Slide 7
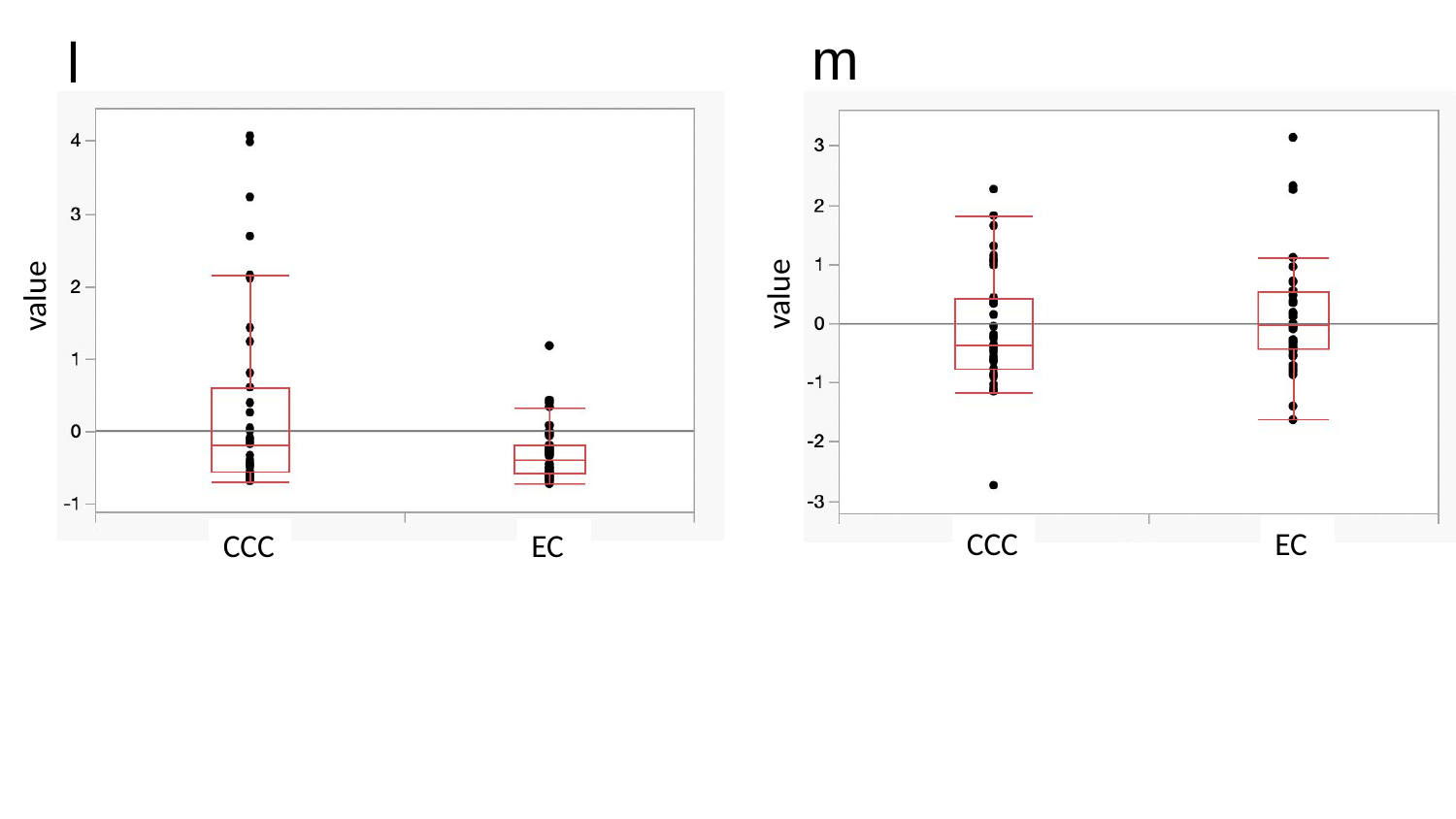

m
l
value
value
CCC
EC
CCC
EC

## Slide 8
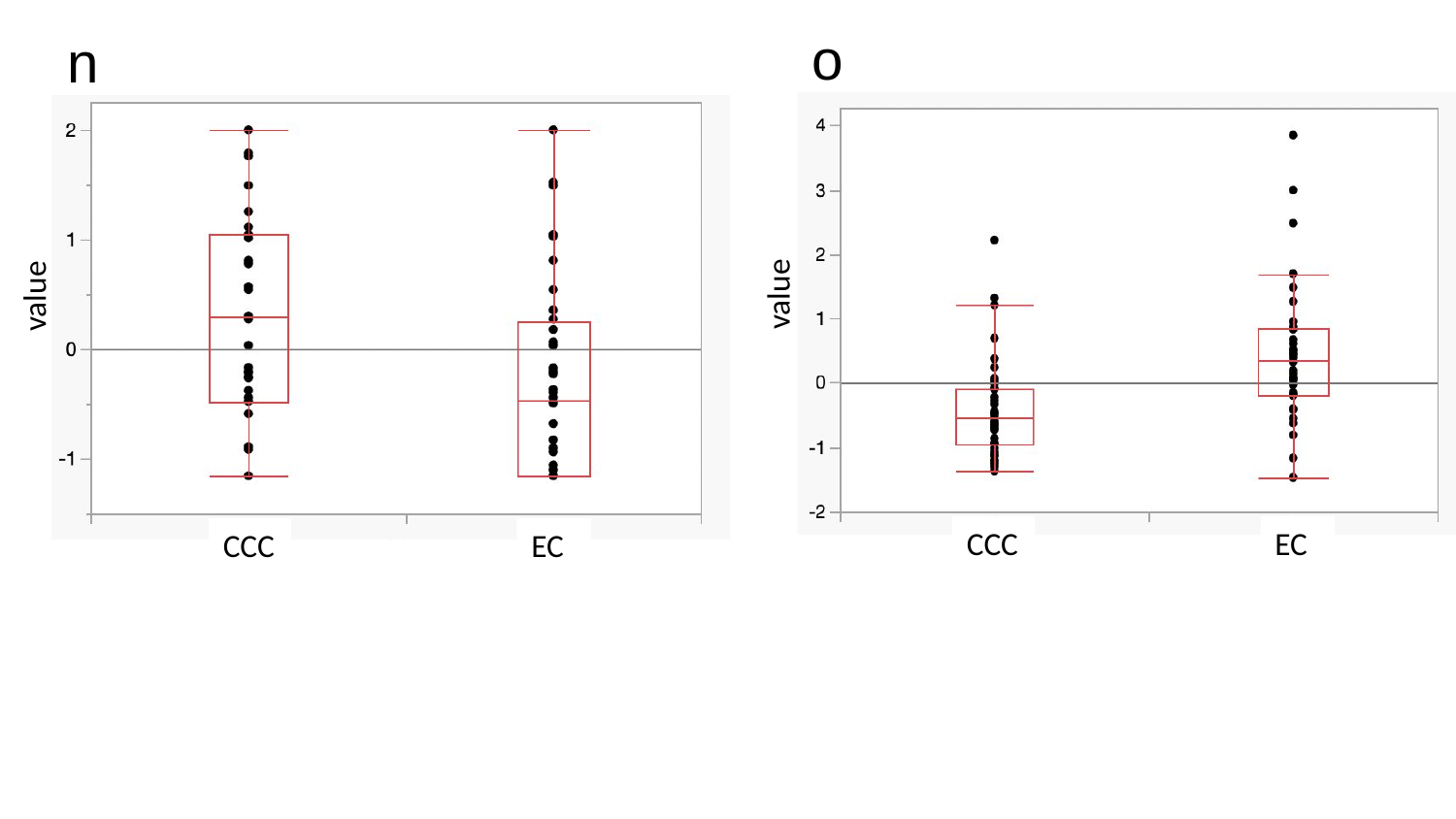

o
n
value
value
CCC
EC
CCC
EC

## Slide 9
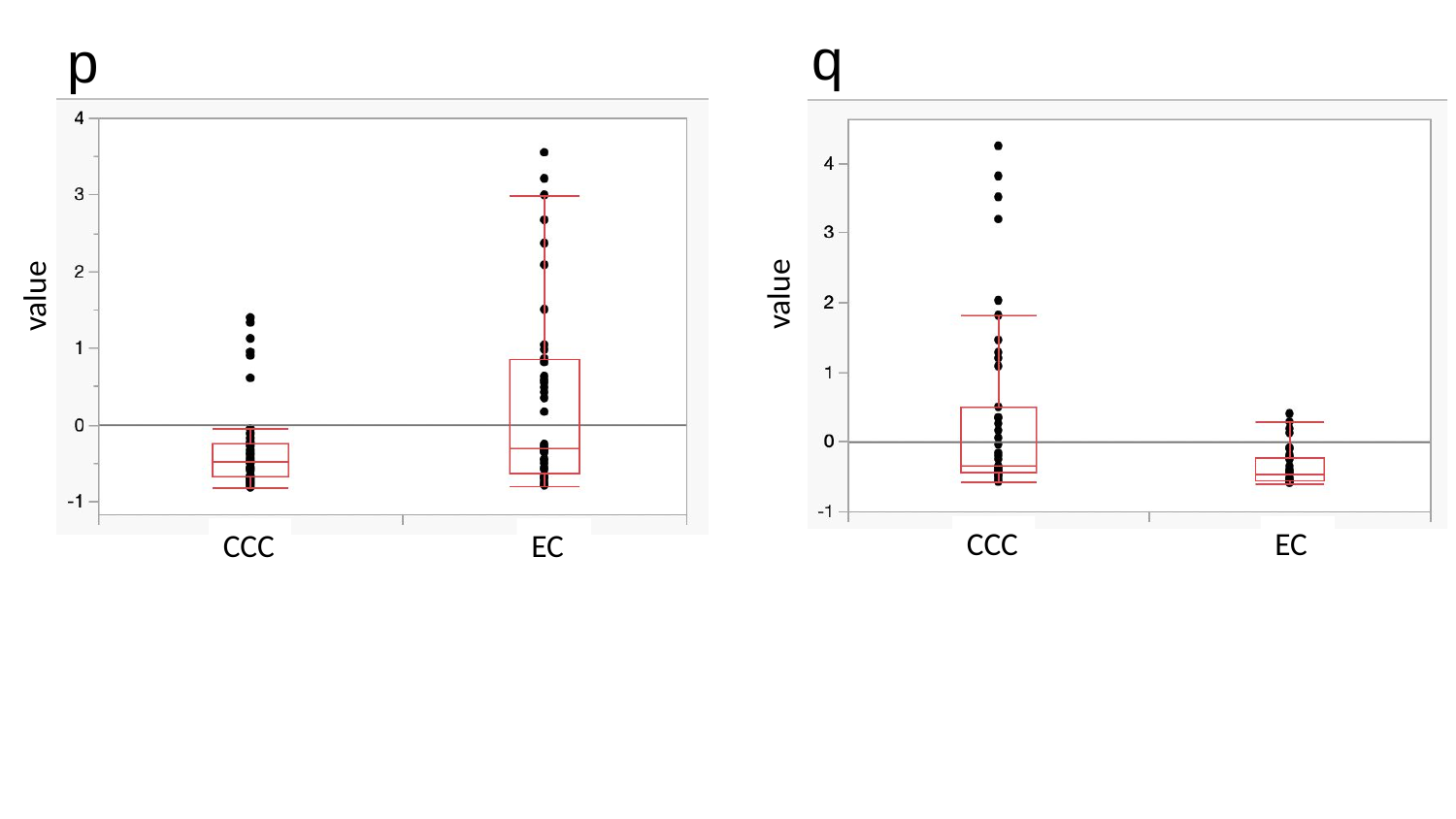

q
p
value
value
CCC
EC
CCC
EC

## Slide 10
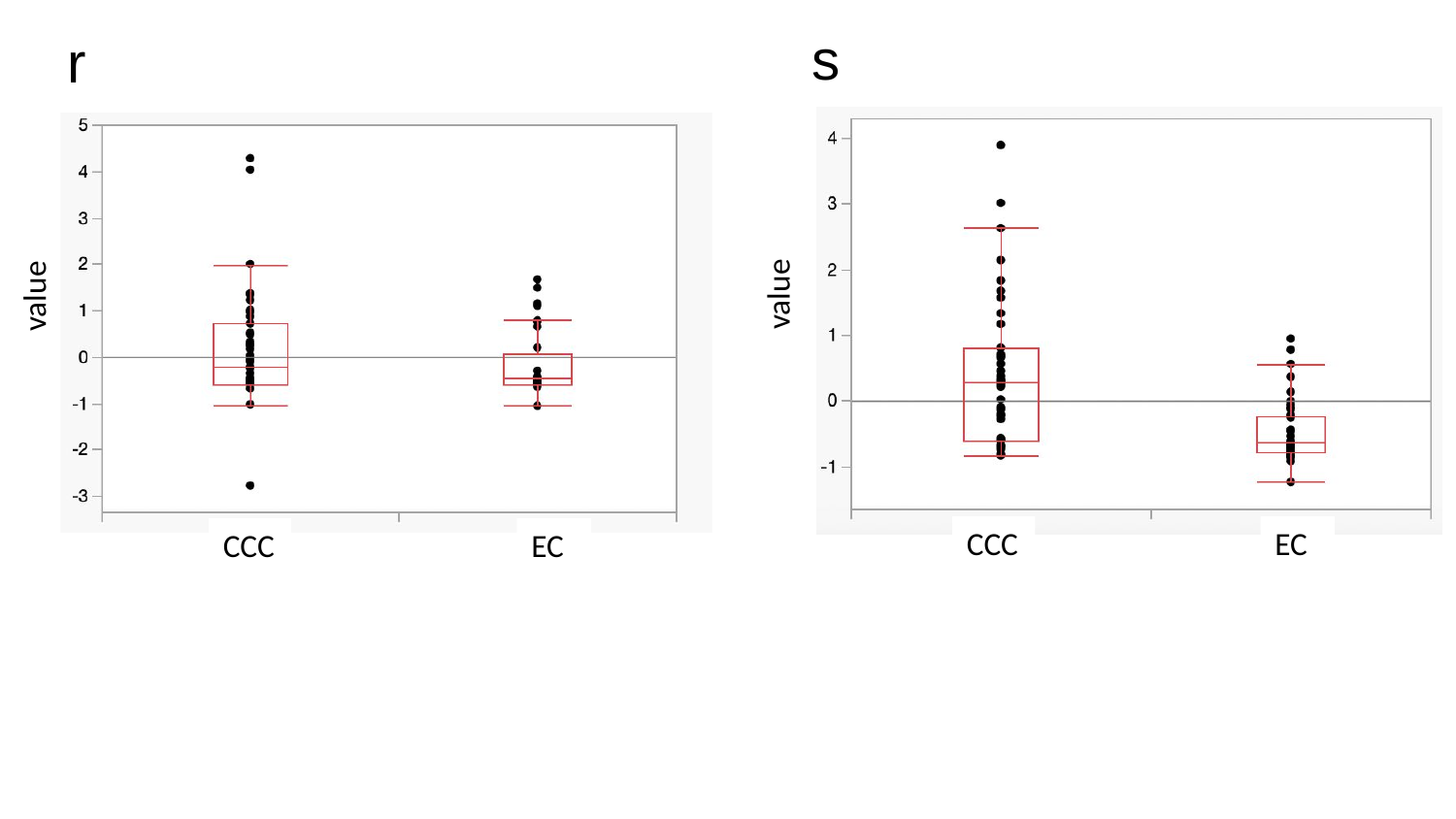

s
r
value
value
CCC
EC
CCC
EC

## Slide 11
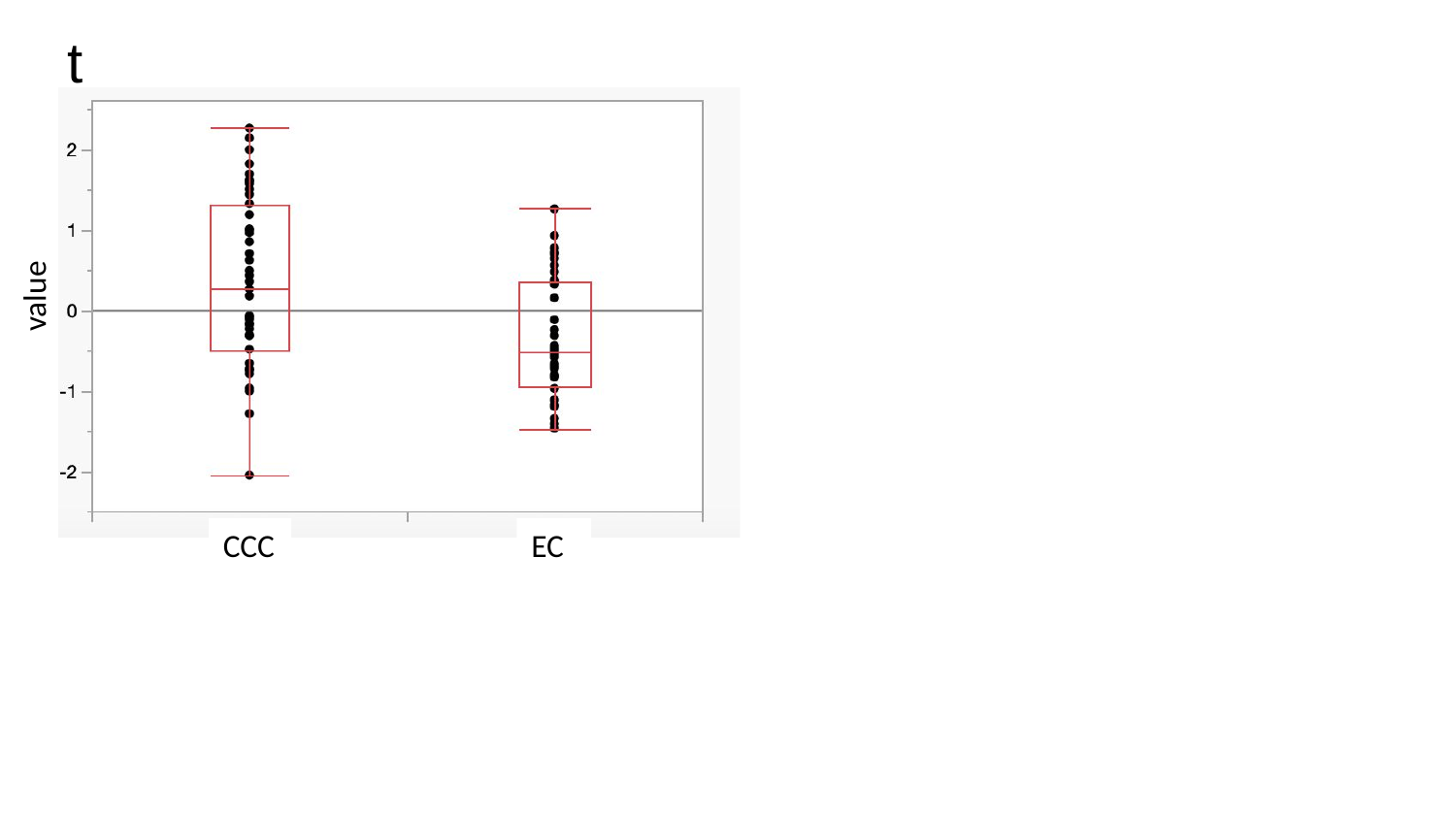

t
value
CCC
EC

## Slide 12
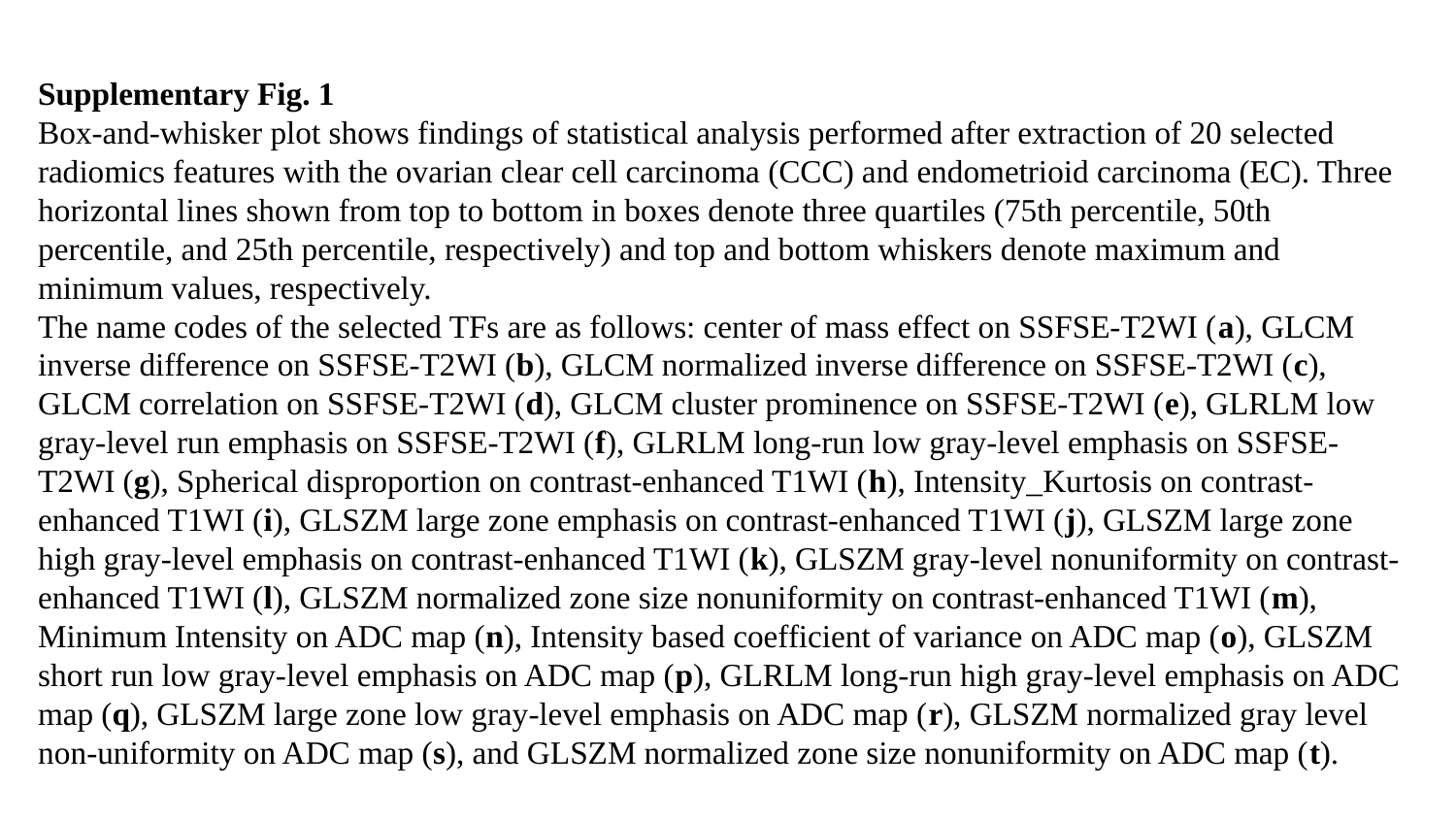

Supplementary Fig. 1
Box-and-whisker plot shows findings of statistical analysis performed after extraction of 20 selected radiomics features with the ovarian clear cell carcinoma (CCC) and endometrioid carcinoma (EC). Three horizontal lines shown from top to bottom in boxes denote three quartiles (75th percentile, 50th percentile, and 25th percentile, respectively) and top and bottom whiskers denote maximum and minimum values, respectively.
The name codes of the selected TFs are as follows: center of mass effect on SSFSE-T2WI (a), GLCM inverse difference on SSFSE-T2WI (b), GLCM normalized inverse difference on SSFSE-T2WI (c), GLCM correlation on SSFSE-T2WI (d), GLCM cluster prominence on SSFSE-T2WI (e), GLRLM low gray-level run emphasis on SSFSE-T2WI (f), GLRLM long-run low gray-level emphasis on SSFSE-T2WI (g), Spherical disproportion on contrast-enhanced T1WI (h), Intensity_Kurtosis on contrast-enhanced T1WI (i), GLSZM large zone emphasis on contrast-enhanced T1WI (j), GLSZM large zone high gray-level emphasis on contrast-enhanced T1WI (k), GLSZM gray-level nonuniformity on contrast-enhanced T1WI (l), GLSZM normalized zone size nonuniformity on contrast-enhanced T1WI (m), Minimum Intensity on ADC map (n), Intensity based coefficient of variance on ADC map (o), GLSZM short run low gray-level emphasis on ADC map (p), GLRLM long-run high gray-level emphasis on ADC map (q), GLSZM large zone low gray-level emphasis on ADC map (r), GLSZM normalized gray level non-uniformity on ADC map (s), and GLSZM normalized zone size nonuniformity on ADC map (t).
