# Supplementary material for: Magnetic resonance imaging-based radiomics analysis of the differential diagnosis of ovarian clear cell carcinoma and endometrioid carcinoma: a retrospective study
Source: Jpn J Radiol. 2024 Mar 12;42(7):731–43. doi: 10.1007/s11604-024-01545-z (PMC11217043; doi:10.1007/s11604-024-01545-z)
Supplement: Supplementary file 2 — Supplementary file2 (DOCX 23 KB) [file 11604_2024_1545_MOESM2_ESM.docx]

**Supplementary Table 1** List of the texture of 98 radiomics features on SSFSE T2-weighted and contrast-enhanced T1-weighted images and ADC map

| Category | Radiomics features |
| --- | --- |
| First-order statistics  (n = 19)  Morphological  (n = 12)  Intensity-histogram  (n = 12/23)  GCLM (n = 23)  GLRLM (n = 11)  NGTDM (n = 5)  GLSZM (n = 16) | mean, variance, skewness, kurtosis, median, minimum, 10 percentile, 50 percentile, 90 percentile, maximum, interquartile range, range, mean absolute deviation, robust mean absolute deviation, median absolute deviation, coefficient of variation, quantile coefficient of dispersion, energy, Root mean square  Volume, Approximate Volume, Surface Area, Surface to Volume Ratio, Compactness1, Compactness 2, Spherical Disproportion, Sphericity, Asphericity, Centre of Mass Shift, Maximum 3D Diameter, Integrated Intensity Mean, Variance, Skewness, Kurtosis, Mean Absolute Deviation, Robust Median  mean, variance, skewness, kurtosis, mean absolute deviation, robust mean absolute deviation, median absolute deviation, coefficient of variation, entropy log 2, uniformity, maximum histogram gradient fray level, minimum histogram gradient gray level  Joint Maximum, Joint Average, Joint Variance, Joint Entropy Log2, Difference average, Difference Variance, Difference Entropy, Sum Average, Sum Variance, Sum Entropy, Angular Second Moment, Contract, Dissimilarity, Inverse Difference, Normalized Inverse Difference, Inverse Difference Moment, Normalized Inverse Difference Moment, Inverse Variance, Correlation, Autocorrelation, Cluster Tendency, Cluster Shade, Cluster Prominence  Short Runs Emphasis, Long Runs Emphasis, Low Grey Level Run emphasis, High Grey Level Run emphasis, Short Run Low Grey Level emphasis, Short Run High Grey Level emphasis, Long Run Low Grey Level emphasis, Long Run High Grey Level emphasis, Grey Level non uniformity, Run Length Nonuniformity, Run Percentage  Coarseness, Contrast, Busyness, Complexity, Strength  Small zone emphasis, Large Zone Emphasis, Low Gray Level Zone Emphasis, High Gray Level Zone Emphasis, Small Zone Low Grey Level Emphasis, Small Zone High Grey Level Emphasis, Large Zone Low Grey Level Emphasis, Large Zone High Grey Level Emphasis, Grey Level non uniformity, Normalized Grey Level non Uniformity, Zone Size non Uniformity, Normalized Zone Size non Uniformity, Zone Percentage, Grey Level Variance, Zone Size Variance, Zone Size Entropy. |

Notes: GLCM, Gray Level Co-occurrence Matrix; GLRLM, Gray Level Run Length Matrix; NGTDM, Neighboring Gray Tone Dependence Matrix; GLSZM, Gray Level Size Zone Matrix
