# Supplementary material for: Magnetic resonance imaging-based radiomics analysis of the differential diagnosis of ovarian clear cell carcinoma and endometrioid carcinoma: a retrospective study
Source: Jpn J Radiol. 2024 Mar 12;42(7):731–43. doi: 10.1007/s11604-024-01545-z (PMC11217043; doi:10.1007/s11604-024-01545-z)
Supplement: Supplementary file 3 — Supplementary file3 (DOCX 21 KB) [file 11604_2024_1545_MOESM3_ESM.docx]

**Supplementary Table 2** Representative 20 radiomics features selected by the LASSO regression analysis

| Features code | CCC (n = 43) | EC (n = 40) | Univariate Analysis (p) | LASSO estimate |
| --- | --- | --- | --- | --- |
| SSFSE-T2WI  Center of mass effect  GLCM inverse difference  GLCM normalized inverse difference  GLCM correlation  GLCM cluster prominence  GLRLM low gray-level run emphasis  GLRLM long-run low gray-level emphasis  Contrast-enhanced T1WIs  Spherical disproportion  Intensity_Kurtosis  GLSZM large zone emphasis.  GLSZM large zone high gray-level emphasis  GLSZM gray-level nonuniformity  GLSZM normalized zone size nonuniformity  ADC map  Minimum Intensity  Intensity based coefficient of variance  GLRLM short-run low gray-level emphasis  GLRLM long-run high gray-level emphasis  GLSZM large zone low gray-level emphasis  GLSZM normalized gray-level nonuniformity  GLSZM normalized zone size nonuniformity | -0.1977 ± 0.8392  -0.0598 ± 1.0760  -0.0589 ± 1.0840  -0.1524 ± 0.9128  -0.1957 ± 0.6302  -0.2540 ± 0.8694  -0.1108 ± 0.9864  -0.4252 ± 0.7753  0.2110 ± 1.1767  0.1712 ± 1.1344  0.2528 ± 1.1344  0.2656 ± 1.2712  -0.1167 ± 0.1255  0.3061 ± 1.0127  -0.4162 ± 0.7479  -0.3034 ± 0.5891  0.3400 ± 1.2821  0.1591 ± 1.2101  0.4216 ± 1.1674  0.3265 ± 1.1000 | 0.2125 ± 1.1201  -0.0642 ± 0.9206  0.0633 ± 0.9106  0.1638 ± 1.0840  0.2103 ± 1.2599  0.2731 ± 1.0681  0.1191 ± 1.0132  0.4571 ± 1.0206  -0.2269 ± 0.7138  -0.1840 ± 1.0883  -0.2718 ± 0.7553  -0.2854 ± 0.4482  0.1254 ± 0.9394  -0.3291 ± 0.8853  0.4474 ± 1.2821  0.3261 ± 1.2322  -0.3655 ± 0.2602  -0.1710 ± 0.6838  -0.4533 ± 0.4776  -0.3510 ± 0.7450 | 0.069  0.469  0.545  0.106  0.276  **0.008**  0.234  **< 0.001**  0.491  0.096  **0.029**  0.082  0.242  **0.005**  **<0.001**  **0.028**  **< 0.001**  0.125  **< 0.001**  **0.002** | -0.0488  -0.0034  -0.1123  -0.1680  -0.1298  -0.1128  -0.4694  -0.6347  0.3092  0.0009  0.6476  0.2384  -0.2800  0.0884  -0.1001  -0.3257  0.1060  0.1043  0.5540  0.0218 |

Notes: MRI, magnetic resonance imaging; EC, Endometrioid carcinoma; CCC, Clear cell carcinoma; SSFSE-T2WI, single shot first spin echo T2-weighted image; T1WI, T1-weighted image; SI, signal intensity; SD, standard deviation; ADC, apparent diffusion coefficient; GLCM, Gray Level Co-occurrence Matrix; GLRLM, Gray Level Run Length Matrix; GLSZM, Gray level Size Zone Matrix.

All 98 texture features (TFs) were included in the LASSO regression analysis. Features with a coefficient other than 0 are shown in this table. Regarding all variables, numeric values were standardized by SPSS ver. 26 before the statistical analysis (* standardized values are shown). ICC intraclass correlation coefficient, LASSO least absolute shrinkage and selection operator, SD standard deviation, CCC clear cell carcinoma, EC endometrioid carcinoma.
