# Supplementary material for: Magnetic resonance imaging-based radiomics analysis of the differential diagnosis of ovarian clear cell carcinoma and endometrioid carcinoma: a retrospective study
Source: Jpn J Radiol. 2024 Mar 12;42(7):731–43. doi: 10.1007/s11604-024-01545-z (PMC11217043; doi:10.1007/s11604-024-01545-z)
Supplement: Supplementary file 4 — Supplementary file4 (DOCX 23 KB) [file 11604_2024_1545_MOESM4_ESM.docx]

**Supplementary Table 3** Representative MR-based features with radiomics analysis-based features selected by the LASSO regression algorithm in the combined model

| Feature name code | LASSO estimate |
| --- | --- |
| Apparent Diffusion Coefficient (MRI-based features)  GLSZM normalized gray-level non-uniformity on ADC map (Radiomics features)  GLSZM large-zone high gray-level emphasis on contrast-enhanced T1WI ((Radiomics features)  Sphericity on contrast-enhanced T1WI (Radiomics features)  Intensity_Range on ADC map (Radiomics features)  Absence of thickening of the uterine endometrium (MRI-based features)  Intensity_Kurtosis on contrast-enhanced T1WI (Radiomics features)  GLSZM large-zone size low-gray level emphasis on ADC map (Radiomics features)  GLRLM long-run low gray-level emphasis on T2WI (Radiomics features)  Center of mass effect on contrast-enhanced T1WI (Radiomics features)  Intensity_Histogram entropy log2 on ADC map (Radiomics features)  GLCM inverse difference on T2WI (Radiomics features)  Spherical disproportion on contrast-enhanced T1WI (Radiomics features) | 0.9868  0.4015  0.3327  0.2441  0.1694  0.1604  0.0879  0.0498  -0.0174  -0.0223  -0.0706  -0.1049  -0.3719 |

Notes: All MRI-based features and texture features were analyzed by the LASSO regression algorithm. Variables with a coefficient other than 0 were selected as representative parameters are demonstrated in this table. All numeric values were standardized before the statistical analysis. LASSO, least absolute shrinkage and selection operator; GLSZM, Gray Level Size Zone Matrix, GLRLM, Gray Level Run Length Matrix; GLCM, Gray Level Co-occurrence Matrix; cT1WI, contrast-enhanced T1-weiguted image; T2WI, T2-weigted image; ADC, apparent diffusion coefficient
